# Modulation of paracrine signaling by CD9 positive small extracellular vesicles mediates cellular growth of androgen deprived prostate cancer

**DOI:** 10.18632/oncotarget.11111

**Published:** 2016-08-08

**Authors:** Carolina Soekmadji, James D. Riches, Pamela J. Russell, Jayde E. Ruelcke, Stephen McPherson, Chenwei Wang, Chris M. Hovens, Niall M. Corcoran, The Australian Prostate Cancer Collaboration BioResource, Michelle M. Hill, Colleen C. Nelson

**Affiliations:** ^1^ Australian Prostate Cancer Research Centre-Queensland, Institute of Health and Biomedical Innovation, School of Biomedical Sciences, Faculty of Health, Queensland University of Technology, Brisbane, Australia; ^2^ Translational Research Institute, Brisbane, Queensland, Australia; ^3^ Central Analytical Research Facility, Institute for Future Environments, Queensland University of Technology, Brisbane, Queensland, Australia; ^4^ The University of Queensland Diamantina Institute, The University of Queensland, Brisbane, Australia; ^5^ Australian Prostate Cancer Research Centre Epworth, and Department of Surgery, University of Melbourne, Australia; ^6^ Australian Prostate Cancer Collaboration (APCC) Bioresource, Brisbane, Queensland, Australia

**Keywords:** prostate cancer, androgen receptor, extracellular vesicles, CD9, exosomes

## Abstract

Proliferation and maintenance of both normal and prostate cancer (PCa) cells is highly regulated by steroid hormones, particularly androgens, and the extracellular environment. Herein, we identify the secretion of CD9 positive extracellular vesicles (EV) by LNCaP and DUCaP PCa cells in response to dihydrotestosterone (DHT) and use nano-LC–MS/MS to identify the proteins present in these EV. Subsequent bioinformatic and pathway analyses of the mass spectrometry data identified pathologically relevant pathways that may be altered by EV contents. Western blot and CD9 EV TR-FIA assay confirmed a specific increase in the amount of CD9 positive EV in DHT-treated LNCaP and DUCaP cells and treatment of cells with EV enriched with CD9 after DHT exposure can induce proliferation in androgen-deprived conditions. siRNA knockdown of endogenous CD9 in LNCaPs reduced cellular proliferation and expression of AR and prostate specific antigen (PSA) however knockdown of AR did not alter CD9 expression, also implicating CD9 as an upstream regulator of AR. Moreover CD9 positive EV were also found to be significantly higher in plasma from prostate cancer patients in comparison with benign prostatic hyperplasia patients. We conclude that CD9 positive EV are involved in mediating paracrine signalling and contributing toward prostate cancer progression.

## INTRODUCTION

Androgenic steroid hormones are important for proliferation and maintenance of both normal prostate and prostate cancer cells. Upon binding with androgens, a nuclear receptor, the androgen receptor (AR) mediates the transcription of genes involved in cellular proliferation and survival [1]. Treatment for prostate cancer has long focused on targeting the activity of the AR [1–3], however prostate cancer is an heterogeneous disease where the underlying mechanisms for progression to castrate resistant prostate cancer are not clearly understood [4].

Cells, including cancer cells, secrete heterogeneous populations of vesicles, collectively referred to as extracellular vesicles (EV) [5–8]. EV are accepted as important mediators for inter-cellular communication due to their ability to actively transport subsets of receptors and genetic information [9]. This ability to transport different proteins, as well as DNA and RNA, has also been linked to disease progression and cancer metastasis [10–12]. EV have been shown to induce a change of tumour fibroblasts to myofibroblasts and promote angiogenesis in the tumour microenvironment [13, 14]. The EV have also shown potential as delivery agents for therapeutic treatments as they are capable of evading the natural immune system with minimum cytotoxic effects [15] as demonstrated in melanoma, where treatment with EVs containing the MAGE3 peptide has shown promising results, with no grade II toxicity [16]. It has also been recently identified that a cell surface proteoglycan, glypican-1 (GPC1), is specifically enriched in pancreatic cancer-cell-derived EV [17], showing that EV may also have potential as cancer biomarkers.

It is currently understood that the exosome isolation methodologies (differential ultracentrifugation or gradient ultracentrifugation with/without 220 nm filtration) purify heterogeneous populations of small EV [18] and several sub-classes of small EV have been defined. Small EV that originate from intracellular budding at the multivesicular bodies (MVBs) have been commonly referred to as exosomes [18, 19]. Exosomes have a double layered membrane with a particular lipid composition rich in sphingomyelin, cholesterol, and glycerophospholipid [20], and contain MVB-associated proteins and RNAs [9, 21]. Another subclass of EV, the ectosomes or microvesicles, are vesicles with diameter between 100 nm to 1 μm that bud from the plasma membrane [5, 7, 22]. Ectosome biogenesis is triggered by plasma membrane activity, including intracellular calcium influx and by ARF-6, and interactions between the cytoskeletal resident proteins actin and myosin [7, 8, 21, 23]. The ectosomes are enriched with phosphatidylserine and the exposure of phosphatidylserine on the cell surface is a characteristic of ectosome secretion [24]. Other members of EV include large vesicle oncosomes, prostasomes, exosome-like vesicles, and virus-like vesicles [5, 25].

The EV marker CD9 is highly expressed in colorectal, breast, endometrial, and prostate cancer [26] and it has often been used as a common marker of exosomes in the past. However, current evidence suggests that CD9 is a more specific EV marker that may be found in a subset of small EV [5, 27]. CD9 is a member of the tetraspannin protein family and is involved in cellular migration, proliferation and adhesion in diverse cell types; it can oligomerise and bind various integrins and tubulin at the cell surface [28]. While antibodies against the CD9 antigen can also induce Ca2+ influx in platelets and stimulate phosphatidylserine exposure at the cell surface [29], CD9 positive EV do not appear to be a member of plasma membrane ectosomes, as they have not been reported to express the common ectosome markers CD35, GPA, CD86, CD47 [5].

Exposure to extracellular stimulants will prompt tumour cells to secrete various factors to the external environment, where they can further contribute towards paracrine signalling and mediate cellular interactions with the tumour microenvironment. In this study, we have characterised the role of androgens specifically DHT, in regulating EV secretion from prostate cancer cells and demonstrate that CD9-enriched EV can modulate cellular proliferation when the availability of androgen is limited as may occur during androgen deprivation therapy. The outcome of these studies demonstrate that CD9 positive EV can mediate paracrine signalling in prostate cancer proliferation in the absence of androgens and that CD9 positive EV are a potential candidate as a prostate cancer biomarker.

## RESULTS

### DHT increases the CD9 positive EV secretion in LNCaP and DUCaP cells

**Figure 1:**
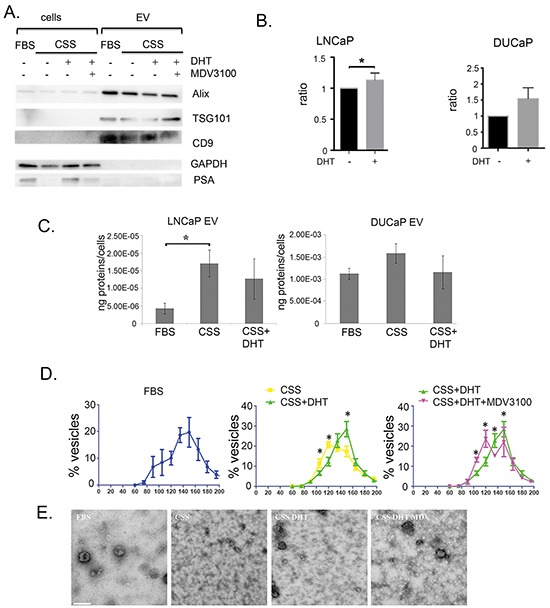
Characterisation of EV in prostate cancer cell lines treated with physiological androgen DHT or under androgen ablation **A**. A representative western blot illustrates the EV markers, Alix, TSG101 and CD9 in vesicles isolated from conditioned medium of LNCaP grown in FBS, CSS (+ EtOH, vehicle) or 10 nM DHT with or without 10 μM MDV3100 (10 μg vesicular proteins or 30 μg cell lysates). Cellular PSA expression was increased by DHT and reduced by MDV3100, used as a marker for AR manipulation. GAPDH was used for loading control of cell lysates. **B**. Treatment with 10 nM DHT showed increased amount of secreted CD9 in conditioned medium of LNCaPs (n = 3, *p<0.05), but not DUCaPs (n = 3). 150 μg proteins of conditioned medium were used for CD9 based TR-FIA assay. **C**. Effect of androgen on the protein yield of secreted vesicles. The secreted vesicles were isolated from conditioned media from LNCaP and DUCaP cells and analysed by BCA assay. Prostate cancer cells were grown in FBS (+ EtOH, vehicle), CSS (+ EtOH, vehicle), or CSS+10 nM DHT. CSS-grown medium increased the amount of secreted vesicular proteins in LNCaP cells (*p<0.05), but not in DUCaP cells. Protein concentrations from each treatment were normalized by cell number (end of experiment) and by protein concentration of vesicles secreted by respective cells in FBS (n = 4, data were represented as mean ± SEM). **D**. Androgen manipulation alters the secretion of LNCaP-derived EV. qNANO measurement and images of vesicles secreted by LNCaP and DUCaP cells. The diameters of isolated vesicles from prostate cancer cells grown in FBS (+ EtOH, vehicle), CSS (+ EtOH), CSS + 10 nM DHT and CSS+10 nM DHT + 10 μM MDV3100 were measured by qNANO using NP100 filter (n = 3-4 biological replicates, minimum of 500 vesicles per measurement, *p<0.05). Data were represented as mean ± SEM. **E**. Representative images captured by Transmission Electron Microscopy of vesicles secreted by LNCaP cells (scale bar: i=200nm). LNCaP cells were grown in FBS (+ EtOH), CSS (+ EtOH), CSS + 10 nM DHT and CSS + 10 nM DHT + 10 μM MDV3100.

Depletion of androgens can suppress the secretion of proteins by AR positive prostate cancer cells, as is seen in secretion of the prostate cancer clinical biomarker prostate specific antigen (PSA) [30]. Small EV were isolated by sequential ultracentrifugation from conditioned media from androgen responsive LNCaP and DUCaP cells in androgen deprived conditions (in CSS), in the presence of the physiological androgen, dihydrotestosterone (DHT) or the AR antagonist MDV3100 (Enzalutamide). We found that secretion of EV is not inhibited by DHT or androgen deprivation as markers of EV Alix, TSG101 and CD9, were detected by western blot in isolated vesicles from LNCaP (Figure 1A and Supplementary Figure S1), and DUCaP (Supplementary Figure S2), irrespective of their treatment. While there was no observed increase or decrease of TSG101 or Alix in EV, the amount of CD9 present in isolated EV was increased when LNCaP and DUCaP cells were treated with DHT (androgen) and was reduced in cells treated with androgen antagonist (MDV3100/Enzalutamide) or when cells were grown in androgen-deprived conditions (in charcoal stripped serum, CSS). The increase of CD9 positive EV in conditioned medium was also shown by measurement using a CD9 TR-FIA assay (Figure 1B).

Although DHT stimulates PSA secretion from cells (Figure 1A), the presence of DHT did not significantly increase the yield of proteins found in EV from LNCaP or DUCaP (Figure 1C), however when LNCaP were cultured in CSS, the yield of EV proteins increased three-fold (Figure 1C, p<0.05), a degree of response that was not observed in DUCaP. Comparatively, when cells were grown in FBS, EVs from DUCaP cells contained 1000-fold higher protein concentration compared to LNCaPs (Figure 1C).

Androgen exposure also induced changes in the diameter of LNCaP-derived EV. The majority of EV isolated from LNCaP grown in FBS or CSS in the presence of DHT (DHT-EV) were approximately 150 nm in diameter, however when grown in androgen-deprived conditions, in CSS (CSS-EV) or in the presence of antagonist MDV3100, there was a significant increase in the population of 120 nm EV concomitant with a reduction in the population of 150 nm EV (Figure 1D). Although similar changes could be observed, in DUCaP cells the change were not significant (Supplementary Figure S2). Representative TEM images show the changes in the size of secreted vesicles from LNCaP upon androgen treatment (Figure 1E), while closer observation showed the EV as cup- or round-shaped vesicles (Supplementary Figure S3).

### Pathway analysis indicates activation of cellular proliferation related pathways in EV secreted by DHT treated cells

**Figure 2:**
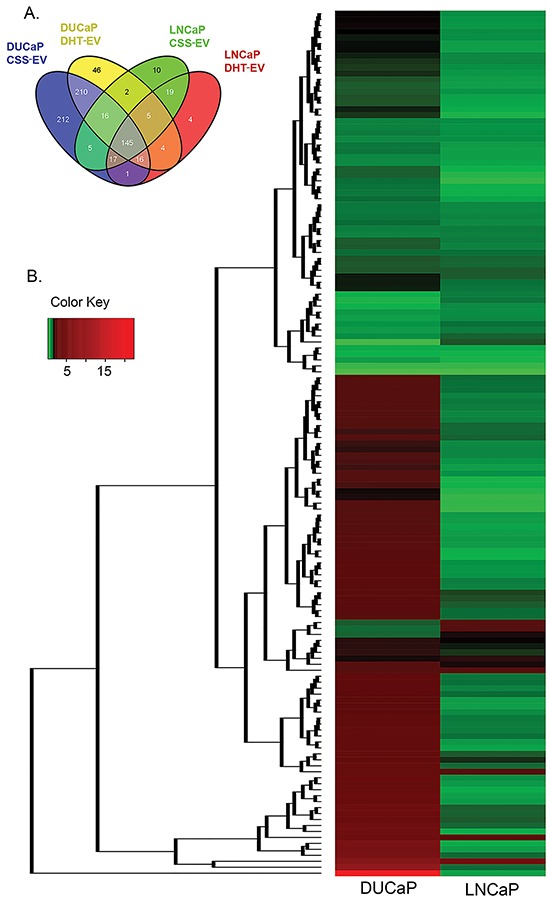
Treatment with DHT changed the relative abundance of vesicular proteins **A**. Venn diagrams visualising the proportion of vesicular proteins found in both LNCaP and DUCaP cells grown in CSS (+ EtOH, vehicle) or CSS + 10 nM DHT. **B**. Unsupervised hierarchical clustering of commonly identified vesicular proteins from both LNCaP and DUCaP cells (n = 145 proteins) based on their ratio of quantified CSS+DHT/CSS (+ EtOH, vehicle) using eMPAI (n = 2-3). Relative protein abundance is colour-coded with red corresponding to a relatively high abundance, green corresponding to a relatively low abundance, and grey indicating missing abundance values.

Using nano-LC–MS/MS following 1D SDS-PAGE separation, we analysed the effects of DHT on the proteome of LNCaP and DUCaP EV and identified a total of 473 and 713 proteins respectively. We did not identify ectosomal (CD35, GPA, CD86 and CD47) or oncosomal (CK18) markers in CSS-EV or DHT-EV from either cell line. For comparative analysis, proteins that were identified in only one out of three biological replicates were not considered for further analysis, leaving a total of 227 and 469 proteins remaining for subsequent analysis for LNCaP and DUCaP EV, respectively. Quantitative analysis was performed using normalized eMPAI ratios for the 145 EV proteins that were commonly found in both cells lines irrespective of treatment (Figure 2A, Supplementary Table S1, with p-value ≤ 0.05 deemed as significant). Unsupervised hierarchical clustering of these proteins illustrated that more than 50% of these proteins were not similarly modified in DHT-treated LNCaP and DUCaP cells (Figure 2B).

**Figure 3:**
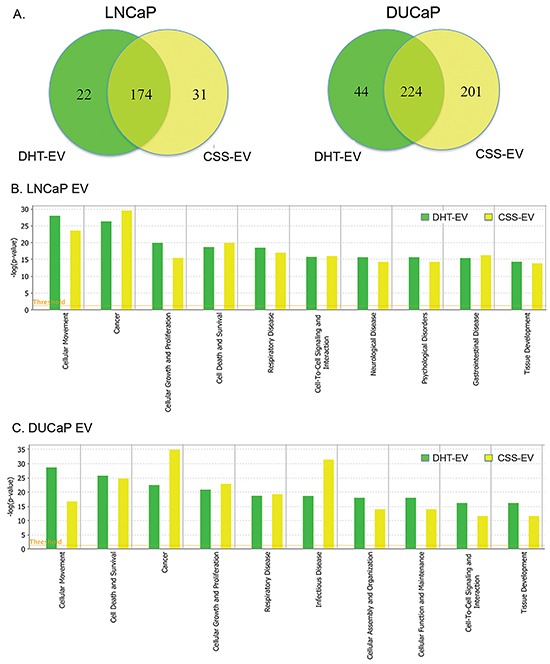
Comparative analysis of vesicular proteins isolated from LNCaP and DUCAP cells **A**. Venn diagrams of proteins identified in at least two biological replicates by mass spectrometry, visualising the proportion of vesicular proteins found in LNCaP or DUCaP when cells were grown in CSS (+ EtOH, vehicle) or CSS+10 nM DHT. **B**. Ingenuity pathway analysis shows the identified “Diseases and Biological Functions” categories of identified proteins in vesicles secreted by LNCaP cells grown in CSS (+ EtOH, vehicle) and after treatment with 10 nM DHT (CSS + 10 nM DHT). **C**. Ingenuity pathway analysis shows the identified “Diseases and Biological Functions” categories of identified proteins in vesicles secreted by DUCaP cells grown in untreated CSS (+ EtOH, vehicle) and after treatment with 10 nM DHT.

In LNCaP cells, 76.6% (174/227) of EV proteins were found in both CSS-EV and DHT-EV, with ∼10% (22/227) and ∼14% (31/227) of the proteins unique to DHT-EV or CSS-EV respectively (Figure 3A). In agreement with previous reports [31, 32], we did not find AR in the list of EV proteins identified by mass spectrometry or by western blot (data not shown). The effect of DHT on DUCaP EV proteins was different to that of LNCaP EV with ∼10% (44/469) of proteins from DUCaP EV uniquely present in DHT-EV, ∼43% (201/469) unique to CSS-EV and 47.8% (224/469 proteins) present in both. To identify potential functional processes and pathways that may be influenced by EV proteins, comparative analysis was performed by sorting the identified functions indicated by Ingenuity Pathway Analysis. We found that the most significantly enriched processes in EV from DHT treated LNCaP and DUCaP cells were ‘cellular movement’, ‘cancer’, ‘cell growth and proliferation’; as well as ‘cell death and survival’ (Figure 3B and 3C, Table 1A). In CSS control cells; ‘cancer’ was the most enriched functional process (Table 1B).

Table 1The effects of androgen manipulation on the composition of LNCaP EV proteins

**Table 1A:** List of 10 most significant pathways in DHT-EV as indicated by IPA

RANK	Categories	Diseases or functions annotation	Molecules	# Molecules
1	Cellular growth and proliferation	proliferation of cells	ADAM15,ADAM9,ARF1,DNAJA1,FGFRL1,HRAS,KIF23,NRAS,PEBP1,PPIA,RRAS,SCAMP4,SCARB1	13
2	Cellular movement	cell movement	ADAM15,ADAM9,ARF1,DNAJA1,HRAS,NRAS,PEBP1,PPAP2A,PPIA,RRAS,SCARB1	11
3	Molecular transport	transport of molecule	ADAM9,ARF1,DNAJA1,HRAS,KPNB1,RAB7A,RAB8A,SCARB1,STXBP3	9
4	Infectious disease	Viral infection	ARF1,DNAJA1,HRAS,KPNB1,LRRC8E,PPIA,RAB7A,RAB8A,SCARB1	9
5	Cellular movement	migration of cells	ADAM15,ADAM9,ARF1,HRAS,NRAS,PPAP2A,PPIA,RRAS,SCARB1	9
6	Cellular development, cellular growth and proliferation	proliferation of tumor cell lines	ADAM15,ADAM9,ARF1,FGFRL1,HRAS,NRAS,PEBP1,SCARB1	8
7	Cancer	carcinoma	ADAM9,ARF1,DNAJA1,HRAS,NRAS,PPAP2A,PPIA,RRAS	8
8	Cellular movement	cell movement of tumor cell lines	ADAM15,ADAM9,ARF1,HRAS,NRAS,PEBP1,PPIA	7
9	Cardiovascular system development and function	development of cardiovascular system	ADAM15,ADAM9,FGFRL1,HRAS,NRAS,PPIA,SCARB1	7
10	Tissue Development	growth of epithelial tissue	ADAM15,HRAS,NRAS,PPIA,RRAS,SCARB1	6

**Table 1B:** List of 10 most significant pathways in CSS-EV as indicated by IPA

RANK	Categories	Diseases or functions annotation	Molecules	# Molecules
1	Cancer	Cancer	ADK,ANXA4,ANXA6,CCT4,CD97,CNDP2,EPHX1,F13A1,FN1,GALK1,H2AFY,ITGA6,ITGB3,PAICS,RALB,RAN,RPLP0,WDR1	18
2	Cell death and survival	cell death	ADK,ANXA4,APOB,CCT4,EPHX1,F13A1,FN1,ITGA6,ITGB3,MDH1,PCBP2,RALB,RAN, RPLP0	14
3	Infectious disease	Viral infection	ADK,ANXA6,APOB,CD97,COL5A1,F13A1,FN1, HLA-C,ITGB3,PACSIN3, PCBP2, RALB,RAN	13
4	Cancer	abdominal neoplasm	ANXA4,CCT4,CD97,F13A1,FN1,H2AFY,ITGA6,ITGB3,MDH1,PAICS,RAN,RPLP0	12
5	Cell death and survival	necrosis	APOB,CCT4,EPHX1,F13A1,FN1,ITGA6,ITGB3,MDH1,PCBP2,RALB,RAN,RPLP0	12
6	Cancer	abdominal cancer	ANXA4,CCT4,F13A1,FN1,H2AFY,ITGA6,ITGB3,PAICS,RAN,RPLP0	10
7	Cell death and survival	apoptosis	ANXA4,CCT4,EPHX1,FN1,ITGA6,ITGB3,MDH1,PCBP2,RALB,RPLP0	10
8	Dermatological diseases and conditions	psoriasis	CD97,EPHX1,F13A1,FN1,H2AFY,HLA-C,ITGA6,PCBP2,RAN	9
9	Organismal survival	organismal death	ADK,APOB,COL5A1,F13A1,FN1,ITGA6,ITGB3,RALB,WDR1	9
10	Cancer	breast or colorectal cancer	ADK,ANXA4,CCT4,CNDP2,FN1,H2AFY,ITGA6,PAICS,WDR1	9

LNCaP is a well characterised prostate cancer cell line, extensively used as a model for androgen response [33]. Using Ingenuity Pathway Analysis (IPA), 103 proteins in LNCaP derived DHT-EV and 99 proteins in CSS-EV were identified as involved in cell proliferation, with 91 proteins in common (Table 2). Thirteen proteins were found uniquely in EV from DHT-treated LNCaP cells, including members of the RAS oncoproteins (HRAS, NRAS, RRAS), Phosphatidic Acid Phosphatase Type 2A (PPAP2A) and the A Disintegrin and Metalloproteinase (ADAM) protein family (ADAM9 and ADAM15), expression of which is regulated by AR [34–37]. The EV markers CD9, TSG101 and Alix (PDCD6IP), were also identified by IPA as part of this cellular proliferation pathway. Quantitative analysis on EV proteins identified by mass spectrometry and analysed by eMPAI normalisation shows that consistent with Western Blot results the EV marker, CD9, was increased by DHT in isolated EV by >1.5-fold, while other MVB-derived EV markers, Alix and TSG101 were not affected (Table 3).

**Table 2:** EV secreted from DHT-treated LNCaP cells indicate the role of EV in cellular proliferation

CSS-EV specific	DHT-EV specific	Common elements in CSS-EV and DHT-EV:					
ADK	ADAM9	A2M	CD59	DNAJA2	ITGB1	PDCD6IP	SERPINF1
CD97	ADAM15	ADAM10	CD81	EEF1A1	ITIH4	PGK1	SLC29A1
FN1	DNAJA1	ADAMTS1	CD151	EIF4A1	JUP	PKM	SLC2A1
H2AFY	FGFRL1	AFP	CD276	ENO1	KRT2	PLG	SLC3A2
ITGA6	HRAS	AHCY	CDC42	EPCAM	KRT10	PLXNB2	ST14
ITGB3	NRAS	AHSG	CFL1	F2	KRT14	PRDX1	STEAP2
RALB	PEBP1	ALB	CLEC11A	F11R	LDHA	PRDX2	TFPI
RAN	PPAP2A	ANXA7	CLIC1	FBLN1	LTF	RAC1	TFRC
	PPIA	ANXA11	CLTC	FLOT1	LUM	RALA	THBS1
	RRAS	APOE	CNP	GNAI1	MARCKSL1	RAP1B	TSG101
	SCAMP4	BSG	COL6A1	GNAS	MFGE8	RHOA	TUBB
	SCARB1	C3	COMP	GNB1	NAP1L1	RNF20	VCL
		C5	CTNNA1	GSN	NRP1	SDCBP	VCP
		C9	CTNNB1	HSP90AB1	PARK7	SELENBP1	VPS28
		CD9	CTNND1	HSPA8	PDCD6	SERPINC1	YWHAG
							YWHAQ

**Table 3:** The relative abundance of identified proteins and their cellular localisation

Symbol	Entrez Gene Name	UniProt/Swiss-Prot Accession	Fold Change (DHT-EV)/(CSS-EV)	Location	Family
FBLN1	fibulin 1	FBLN1_HUMAN	−2.308	Extracellular Space	other
F11R	F11 receptor	JAM1_HUMAN	−1.901	Plasma Membrane	other
SELENBP1	selenium binding protein 1	SBP1_HUMAN	−1.624	Cytoplasm	other
YWHAQ	tyrosine 3-monooxygenase/tryptophan 5-monooxygenase activation protein, theta polypeptide	1433T_HUMAN	−1.587	Cytoplasm	other
CD9	CD9 molecule	CD9_HUMAN	1.530	Plasma Membrane	other
KRT10	keratin 10	K1C10_HUMAN	2.038	Cytoplasm	other
RRAS	related RAS viral (r-ras) oncogene homolog	RRAS_HUMAN	D	Cytoplasm	enzyme
ADAM15	ADAM metallopeptidase domain 15	ADA15_HUMAN	D	Plasma Membrane	peptidase
ADAM9	ADAM metallopeptidase domain 9	ADAM9_HUMAN	D	Plasma Membrane	peptidase
NRAS	neuroblastoma RAS viral (v-ras) oncogene homolog	RASN_HUMAN	D	Plasma Membrane	phosphatase
PPAP2A	phosphatidic acid phosphatase type 2A	LPP1_HUMAN	D	Plasma Membrane	enzyme

The amount of proteins is normalised by eMPAI method. The fold change is calculated by normalising the average of quantified DHT-EV proteins (n=2-3) with the average of quantified CSS-EV proteins (+ EtOH, vehicle, n=2-3).

D: specifically found in DHT-EV

### CD9 EV is higher in plasma prostate cancer

**Figure 4:**
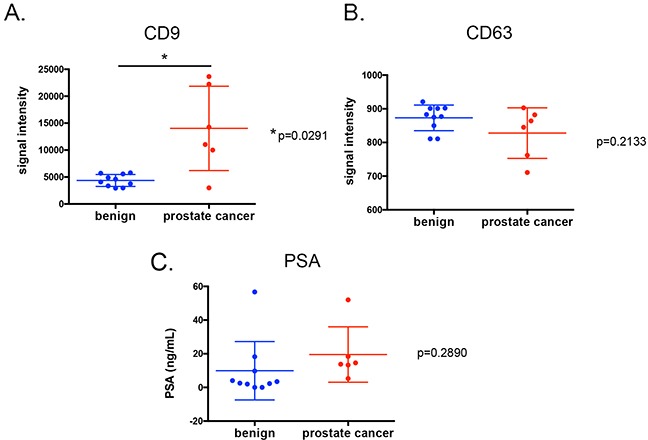
CD9 **(A.)** and CD63 positive EV assays **(B.)** as well as PSA levels **(C.)** on plasma samples from men with prostate cancer (n=6) or diagnosed with benign prostate hyperplasia (n=10). Each point represents an average of duplicate measurements. Variance was estimated with the standard deviation of means for each groups.

The highly sensitive time resolved-fluorescence immunoassay (TR-FIA) for capture/detection of CD9 EV and CD63 EV has been previously employed to assess the content of CD9 positive and CD63 positive EV in urine of prostate cancer patients [38], where levels of CD9 and CD63 positive EV were found to be higher in urine samples from prostate cancer patients. EV can mediate cancer progression by delivering information to distant cells by exploiting the systemic blood circulation, as seen in murine xenografts [39]. EV can also serve as biomarkers for diseases such as cancer [40, 41]. To assess whether CD9 EV can serve as biomarker in prostate cancer, we examined the level of CD9 positive and CD63 positive EV in prostate cancer in plasma derived from prostate cancer patients (n=6) and benign prostate hyperplasia (BPH) patients (n=10). Patient characteristics are shown in Table 4. Higher CD9 positive EV were evident in in prostate cancer patients compared to men diagnosed with BPH (Figure 4A), while measurement of CD63 positive EV and PSA did not show significant differences between the cohorts (Figure 4B and 4C).

**Table 4:** Clinical characteristics of patients included in this study

**Men with prostate cancer (n=6)**	**n=6**
Age	67.7 (63-76)
PSA	28.4 (5.3-67)
Gleason score	
7	n=1
9	n=5
**Men diagnosed with benign prostate hyperplasia**	**n=10**
Age	74.3 (65-79)
PSA	8.6 (0-45)

### CD9 enriched EV increased cellular proliferation in androgen deprived LNCaP cells

**Figure 5:**
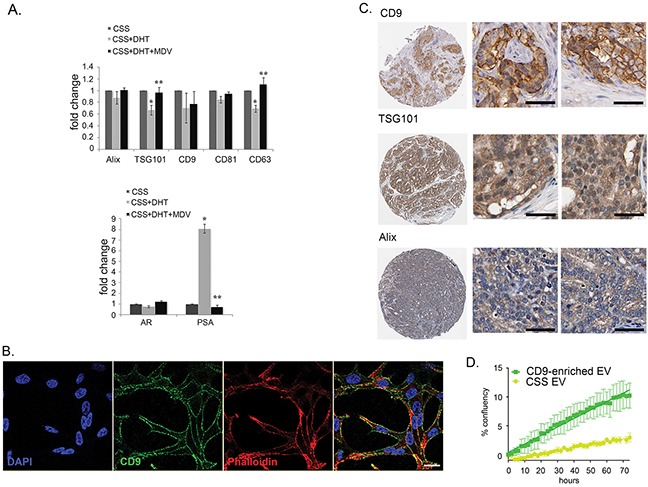
CD9 expression in LNCaP and tissue samples **A**. TSG101 and CD63 mRNA levels dropped in LNCaP cells treated with 10 nM DHT (*p<0.05), and increased after treatment with 10 μM of androgen receptor antagonist drug, MDV3100 (**p<0.05). AR expression was not changed across the treatment, while the level of PSA was increased up to eight-fold in DHT-treated cells and reduced by MDV3100. Gene expression was normalized to the housekeeping gene rpl32, and then expressed relative to the vehicle control (EtOH) at the same time point. Data were analysed with SDS 2.3 software using 2-ΔΔCt (n = 4). Data were represented as mean ± SEM. **B**. Subcellular localisation of CD9 in LNCaP cells imaged by confocal. In the overlay image (right panel), CD9: green, cytoskeletal marker F-actin (labelled by phalloidin): red, nucleus marker DAPI: blue. Potential co-localisation between CD9 and F-actin is in yellow. Scale bar: i = 20 μm. **C**. Representative image of immunohistochemistry staining (brown) in a high-grade prostatic adenocarcinomas for CD9 (CAB002490, Male, age 70, Prostate (T-77100), Adenocarcinoma, High grade (M-814033), Patient id: 3191);TSG101 (HPA006161, Male, age 64, Prostate (T-77100), Adenocarcinoma, High grade (M-814033), Patient id: 250); and Alix (HPA011905, Male, age 61, Prostate (T-77100), Adenocarcinoma, High grade (M-814033), Patient id: 3486). Scale bar: i = 100 μm. **D**. Treatment of CSS-grown LNCaP cells with CD9-enriched EV isolated from LNCaP cells increased the rate of proliferation of androgen-deprived LNCaP cells. Data were represented as mean ± SEM.

We further questioned whether CD9 expression and secretion in EV is a result of the activation of AR by DHT. It has been reported that CD9 expression was increased in LNCaP cells treated with a synthetic androgen, R1881 at 10 nM [42], however, we found that at a more physiologically relevant concentration of 10 nM DHT, CD9 mRNA levels did not increase in LNCaP cells (Figure 5A). Androgen manipulation also did not alter the CD9 cellular protein levels (data not shown). In contrast, levels of TSG101 and CD63 in LNCaP were significantly reduced by treatment with 10 nM DHT (∼30% reduction). This reduction was antagonized by the presence of 10 μM MDV3100 (Figure 5A).

While it is currently accepted that cells secrete various populations of EV and that CD9 could be a marker of a distinct population of small EV [43], the EV contents are dependent on the cellular context [5, 23, 27, 44]. CD9 has also been reported to be localised at the plasma membrane in various cell lines [5]. We further investigated the differences on EV markers CD9, TSG101 and Alix in prostate cancer cell lines and in prostate tissue. Irrespective of treatment, we found that CD9 localised at the plasma membrane in LNCaP (Figure 5B) and that CD9 is also found localise predominantly at the plasma membrane in human prostate adenocarcinoma tissue samples (Figure 5C, www.proteinatlas.org; [26]). In contrast, the regulators of MVB-derived exosome biogenesis, TSG101 and Alix, are both found ubiquitously in human prostate adenocarcinoma (Figure 5C), with strong expression in the cytoplasm of human prostate cancer specimens, and in the cytosol of LNCaP (data not shown).

Pathway analysis indicated that DHT-induced secretion of EV may be capable of influencing cell proliferation. We investigated whether EV from CD9-enriched LNCaP cells could be involved in modulating the proliferation of LNCaP cells grown under androgen-deprived conditions. When grown in CSS, the confluency of LNCaP cells treated with CD9-enriched EV increased by 5.3-fold in comparison with LNCaP cells treated with control CSS-EV (p<0.0001, in 48h, Figure 5D), implying that CD9-enriched EV can promote growth of androgen-deprived LNCaP cells. Steroid hormones, such as DHT, are not retained inside vesicles, as they are able to diffuse through cell membranes [45]. The observed DHT from treated EV was confirmed by ELISA measurement (data not shown).

**Figure 6:**
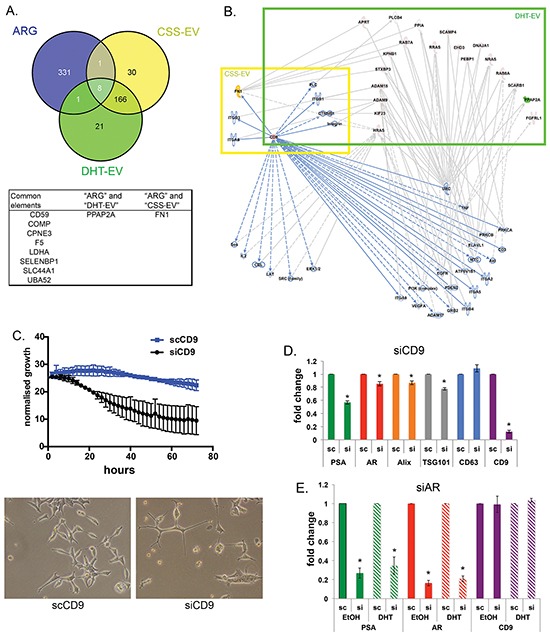
CD9 is an EV-derived regulator for prostate cancer proliferation **A**. Venn diagram shows AR-regulated genes and EV proteins isolated from CSS and CSS DHT cultured LNCaP cells. **B**. Pathway analysis illustrates the interactions of CD9, CSS-EV and DHT-EV proteins. Pathway analysis identified CD9 could be the upstream regulator for DHT-EV content through various cancer related pathways. FN1 is highlighted in yellow and PPAP2A in green. **C**. Knockdown of CD9 using siRNA reduced the cellular growth of LNCaP cells. LNCaP cells were treated with the indicated concentrations (5 nM) of siRNA or control (scRNA), and growth as a function of confluence was measured after 24 h in real-time by phase contrast microscopy on an IncuCyte HD system continuously for 72 h (n = 3, mean ± SE). Representative images of siRNA treated LNCaPs were taken after 48 h addition of siRNA. **D**. Knockdown CD9 reduced the mRNA expression of TSG101, Alix, as well as AR, and PSA, RNA samples were harvested 48 h after treatment with 5 nM CD9 siRNA. Gene expression was normalized to the housekeeping gene rpl32, and then expressed relative to the scRNA. Data were analysed with SDS 2.3 software using 2-ΔΔCt (n = 4-5, *p<0.05), represented as mean ± SEM. **E**. Knockdown AR reduced the mRNA expression of the AR classical regulated gene PSA as expected, but did not alter the mRNA level of CD9. RNA samples were harvested 72 h after treatment with 10 nM AR siRNA on cells grown in CSS treated with 10 nM DHT or EtOH (vehicle). Gene expression was normalized to the housekeeping gene rpl32, and then expressed relative to the scRNA. Data were analysed with SDS 2.3 software using 2-ΔΔCt (n = 3-4, *p<0.05), represented as mean ± SEM.

To investigate whether the AR plays a major role in altering EV protein content upon DHT treatment, we compared our EV data with in-house microarray gene expression profiling on DHT-treated LNCaP cells. We compiled a list of 341 genes identified whose expression is known to be increased/decreased by AR, the AR Regulated Genes (ARG, Supplementary Table S2). Comparing our mass spec data with the ARG list, we identified 10 vesicular proteins whose expression may be directly regulated by AR (Figure 6A), implicating AR in determining at least some of the proteins in secreted EV. Two proteins known to be regulated by AR were identified in EV: Fibronectin 1 (FN1) was found in CSS-EV and PPAP2A was found in DHT-EV (Figure 6A, Supplementary Table S2). Pathway analysis using IPA also indicated the association of CD9 with other proteins such as the oncoproteins of the RAS family, as well as the PPAP2A and FN1 (Figure 6B). The phosphatidic acid phosphatase 2a (PPAP2A/PAP-2a) is involved in *de novo* synthesis of glycerolipids by converting phosphatidic acid to diacylglycerol [36]. FN1 has been used as a marker for cellular motility and shown to inhibit proliferation in AR negative PC3 cells [46–48]. FN1 has also been reported to bind to CD9 and their interaction inhibits cell adhesion to fibronectin [49, 50], which may be indicative of the role of EV-derived FN1 and CD9 in this process.

The role of endogenous CD9 in cellular proliferation appears to depend on cellular context; it has been reported to activate EGFR signalling pathways (pro-proliferative) in gastrointestinal cancer cells [28], but to be anti-proliferative in human colon carcinoma [51]. We examined the proliferative role of CD9 in LNCaP by siRNA driven knockdown of endogenous CD9 (Figure 6C), which resulted in a significant reduction in cell growth. Interestingly siRNA driven knockdown of CD9 also reduced the expression of mRNA for AR, the AR-regulated gene PSA, and the EV markers TSG101 and Alix (Figure 6D, n=4-5, p<0.05), which suggests that CD9 may have a key role in modulating AR activity and the secretion of MVB-derived EV activated by Alix and TSG101. Interestingly, knockdown of endogenous AR, while reducing the mRNA expression of AR and PSA, with or without the presence of DHT, did not alter the expression of CD9 mRNA (Figure 6E, n=3, p<0.05), suggesting a role of CD9 upstream of the AR signalling axis.

## DISCUSSION

The role of androgens in prostate cancer proliferation and progression is widely documented [30, 52, 53]. In this study, for the first time, we report the effect of the physiological androgen, DHT, on EV secretion and protein content in androgen responsive LNCaP and DUCaP prostate cancer cells. Treatment with CD9 enriched EV was able to increase the cellular proliferation of androgen-deprived LNCaP cells independently of DHT, thereby demonstrating a role of CD9 through EV in prostate cancer cell proliferation. We also found that the content of CD9 positive EV in plasma of prostate cancer patients was higher than for BPH patients, warranting further analysis in larger patient cohorts to validate plasma derived CD9 positive EV as potential prostate cancer biomarkers.

Treatment of serum by charcoal-stripping, eliminates serum-derived androgens and other small molecules, resulting in cells becoming more sensitive towards androgens [54] however it has also been shown that prolonged LNCaP culture in androgen-deprived media can cause the cells to become androgen-independent, while still expressing unchanged amounts of AR [55]. While androgen manipulation did not alter the expression of CD9; TSG101 expression was reduced by DHT treatment, and androgen deprivation increased its expression. We did not identify known ectosome markers in our isolated EV, however, CD9 in LNCaP was primarily localised at the plasma membrane, where the biogenesis of ectosomes is described to occur [23], confirming reports by others that CD9 EV is distinct from TSG101 and Alix positive EV [18, 43].

The function of CD9 seems to be dynamic, it can interact with CD63, a tetraspanin MVB-localised protein in platelets [56], suggesting that the CD9 can interact with MVB in other cells [8, 22]. Furthermore, CD9 knockdown reduced the expression of TSG101 and Alix (Figure 6D), well-characterized regulators of MVB-associated EV secretion [5, 57–60]. The EV regulator TSG101 has been reported to play a role in prostate cancer as abnormal transcripts of TSG101 have been identified [61]. TSG101 has also been reported to have a role in AR signaling that appears to be dependent on recruitment of apoptosis-antagonizing transcription factor (AATF) to the AR [62]. It has been proposed that TSG101 controls the level of AR ubiquitination to stabilise AR, allowing AR-regulated transcription in rat-1 and MCF-7 cells [63]. However in contrast, TSG101 has also been reported to repress AR in monkey kidney CV1 cells [64]. While these reports confirm dynamic interactions between TSG101 and AR, the role of TSG101 in AR signalling may be temporal in prostate cancer.

Exposure to DHT enriched for the presence of CD9 in vesicles and reversed the size of small EV population back to 150 nm in diameter in LNCaPs, but not DUCaPs, further suggesting that androgens are involved in pathway(s) involved in EV biogenesis, in particular production of CD9 positive EV in LNCaP cells. It is possible that the shift in size of EV was not detected in DUCaP cells due to the presence of viral particles produced by the cells. The DUCaP cells were derived from the dura mater of a prostate cancer patient and then were established by culturing isolated cancer cells in a mouse xenograft [65]. It has been reported that such practices can infect human-derived cancer cells with viral particles, such as the replication competent murine gammaretroviruses [66]. Viral particles can also be co-secreted with exosomes as seen in 22Rv1 prostate cancer cells [67]. We confirmed that DUCaP cells were positive for MuLV-like viral infection (Supplementary Figure S4). Due to their similar sizes, current methodologies using ultracentrifugation and filtration are not able to separate viral particles from EV [66, 68]. The presence of viral particles can also be detected through the presence of viral RNA in EV, as we observed in EV from DUCaPs; while in contrast, LNCaP cells and LNCaP-derived EV were both shown to be negative for viral contamination (Supplementary Figure S4). A report has also shown that the envelope of HIV-1 virus contains common EV markers, CD9 and CD63 [69] and that EV can exploit the viral entry pathways for uptake in recipient cells [70]. To what extent the viral and EV pathways can overlap in prostate cancer cells which were infected by viral particles is not clear [71] and requires further investigation. Nevertheless, in both LNCaP and DUCaP cell lines, treatment with DHT increased the presence of CD9 in total isolated EV.

In prostate cancer, CD9 has been identified as a candidate gene involved in androgen-deprived cell proliferation through its interaction with IGSF8 [72], suggesting a role of CD9 itself in AR-mediated prostate cancer progression. However we found that knockdown of AR did not alter the expression of CD9, indicating that CD9 is not a classical AR-regulated gene. In urine, the level of CD9 positive and CD63 positive EV have been shown to be higher in men with prostate cancer [73], as well as in plasma (Figure 4), supporting the clinical utility of CD9 positive EV measurement in patients biofluids for men with prostate cancer. Our study has confirmed that DHT can increase the secretion of CD9 positive EV and DHT alters EV content and that CD9 positive EV measurement in patients’ blood based-biofluids may provide alternative biomarkers for prostate cancer patients.

## MATERIALS AND METHODS

### Cell culture

LNCaP (from ATCC) and fibroblast-free DUCaP cells (from Dr Matthias Nees, VTT Technical Research Centre of Finland, Supplementary Figure S2C) were routinely cultured in phenol red-free RPMI 1640 (Invitrogen, Melbourne, Australia) supplemented with 5% foetal calf serum (FBS; Hyclone, Thermo Scientific, Scoresby, Australia). Cellular responses to androgens were assessed by culturing cells in 150 mm plates in RPMI + 5% FBS for 72 h. Media were then replaced with RPMI + 5% charcoal-stripped serum (CSS; Sigma, St Louis, US) for 48 hours after which media were replaced with fresh RPMI + 5% vesicle-depleted CSS + 20% EtOH (vehicle) or 10 nM dihydrotestosterone (DHT) with or without an androgen antagonist 10 μM MDV3100 (Enzalutamide) for 48 h. For FBS control cells, after 120 h, medium was replaced with fresh RPMI + 5% vesicle-depleted FBS. Vesicle-depleted serum was prepared by ultracentrifugation of 20% serum at 100,000 xg overnight followed by filtration using a 0.22 μm vacuum filter (Invitrogen, Melbourne, Australia). All chemicals and reagents are obtained from Sigma, unless indicated otherwise.

### Isolation of EV

Small extracellular vesicles were prepared from the conditioned medium by a series of differential centrifugation steps, as described [74]. The conditioned medium was harvested, centrifuged at 2000xg for 20 min to eliminate cells and dead cells, at 10,000xg for 30 min to eliminate cell debris, followed by ultracentrifugation at 100,000xg for 2 h to pellet EV. The EV pellet was washed once in PBS and spun at 100,000xg for 1.5 hour. EV were then resuspended in PBS for further experiments or resuspend in 0.5% depleted CSS for functional analysis and stored at −80°C until analysis. Protein content was measured using the BCA protein assay kit (Pierce, Rockford, US).

### Western blot

Cells were lysed at the end of the experiment in 10 mM Tris pH 7.8, 1 mM EDTA, 150 mM NaCl, 1% NP40, cOmplete protease inhibitor cocktail EDTA-free (Roche, Basel, Switzerland) on ice for 15 min and then spun for 15 min at 10,000xg 4°C to eliminate cellular debris. Isolated EV samples (10 μg protein in PBS) or cell lysate (30 μg protein) were analysed using Trans-Blot Turbo Transfer System (Biorad, Berkeley, USA). Membranes were probed with primary antibodies TSG101 (1:1,000; BD Biosciences), Alix (1: 1,000, Cell Signalling, Danvers, USA), CD9 (1:250; Santa Cruz, Dallas, USA), CHC (1: 200; Abcam, Cambridge, UK; secondary antibody, HRP-conjugated donkey anti-rabbit IgG or HRP-conjugated donkey anti-mouse IgG (1: 10,000; Millipore, Darmstadt, Germany) and visualized using the ChemiDoc™ MP System.

### Transmission electron microscopy

Isolated EV were fixed with equal volume of 3% glutaraldehyde in 0.1 M sodium cacodylate buffer for 15 min at room temperature, and loaded onto carbon-coated copper grids. Samples were negatively stained with 1% aqueous uranyl acetate, and observed using a JEOL JEM-1400 Transmission Electron Microscope, at an accelerating voltage of 80kV. Images were acquired on a TVIPS F416 16MP CCD camera.

### Vesicle measurement

The diameter of EV was measured by Tunable Resistive Pulse Sensing (TRPS) technology (qNANO, IZON, Christchurch, New Zealand) using an NP100 nanopore filter and 115 nm polyethylene glycol standard beads. Data were analysed using IZON Control Suite v2.2.64 and frequency distribution of vesicle diameter was analysed using Excel with 15 nm bin interval. Statistical analysis was performed using GraphPad Prism 5 v5.03. Significant differences in the frequency of EV between groups were identified with p ≤ 0.05 (2-way ANOVA Bonferroni post-test, n=3-4).

### Mass spectrometry

Three independent biological replicates of EV (25 μg) from LNCaP and DUCaP treated with DHT (DHT-EV) or control sample (CSS-EV, grown in CSS + EtOH (vehicle)) were processed by SDS-PAGE fractionation, liquid handler-assisted in-gel digest and LC-MS/MS as described [75].

Extracted data were searched against the SwissProt human database (release-2013_05) with fixed carbamidomethylated cysteine and variable oxidized methionine modifications using Spectrum Mill (Agilent, B.04.00.127). The parameters were: up to two missed tryptic cleavages, precursor tolerance 20ppm, product mass tolerance 50ppm, protein score > 11, peptide score > 10, scored peak intensity > 60%. The global false discovery rate was less than 0.5%. Scaffold v4.3.0 was utilised for relative quantification and analysis of Gene Ontology Terms. Protein probabilities were verified using the Protein Prophet algorithm [76]. T-Test statistical analysis was performed on quantitative values normalised using the eMPAI method [77].

Proteins identified only once were excluded from further pathway analysis using QIAGEN’s Ingenuity® Pathway Analysis (IPA®, QIAGEN Redwood City, www.qiagen.com/ingenuity). A non-supervised hierarchical clustering was performed on common proteins found in at least two samples of each treatment across treatments. The ratio of (DHT-EV) / (CSS-EV) data were log2 transformed calculated; and hierarchical clustering was applied to the ratio values via the heatmap.2 function of the plots package in R.

### Detection of murine leukaemia virus-related virus

Detection of murine virus was conducted using the nested PCR protocol and primers previously used to detect Xenotropic Murine leukaemia virus-related virus (XMRV) contamination in prostate cancer samples [78].

### Cell proliferation assay and functional analysis of EV

Live cell imaging was used to monitor changes in cell growth responses following treatment with DHT or EV. Cells were seeded in 96-well plates in RPMI + 5% FBS in RPMI 1640 at 3.0 × 10^3^ and grown to 15-20% confluence. Media was replaced with fresh RPMI + 5% CSS for 48 h and then replaced with fresh RPMI 1640 + 5% CSS or +10 nM DHT (CSS DHT) or CSS (+ EtOH, vehicle), or for functional study, in RPMI 1640 + 0.5% vesicle-depleted CSS in fresh RPMI 1640 + 0.25 mg/ml of isolated vesicles. Growth as a function of increasing confluence was measured in real-time by phase contrast microscopy with the IncuCyte HD system (Essen BioScience), as previously described [79]. Images were taken with a 10x objective at 2 h intervals from three independent experiments, and mean ± standard error measurement (SE) of confluence percentages was computed. Data were analysed and linear regression and statistical analysis were calculated using GraphPad Prism 5 v5.03.

### Transfection of small interfering RNA

To knock down endogenous CD9 expression, CD9-specific small interfering RNA (CD9 siRNA) was generated using published sequence ([80], 5′-GAGCATCTTCGAGCAAGAA-3′). Cells were seeded at 9 × 10^4^ in six-well plates in RPMI + 5% FBS in RPMI 1640, and transfected with 5 nM CD9 siRNA after 72 hours with Lipofectamine RNAiMax (Invitrogen). Scrambled siRNA (CD9 scRNA; 5′-GGGAAUCGCCCAAAUAGAU-3′) was used as a negative control. Cellular proliferation was observed using IncuCyte HD system as described above 24 h after transfection. Forty-eight hours after transfection, cells were analyzed for knockdown efficiency by qRT PCR. To knock down endogenous AR expression, ON-TARGET plus AR siRNA (J-003400-07-0005, Thermo Scientific) was transfected on LNCaP grown in CSS for 24 h. Cells were treated with 10 nm AR siRNA or scRNA (D-001810-02) with Lipofectamine (Invitrogen) for 24 h, media were replaced and treated with 10 nM DHT or EtOH (vehicle) for 48 h.

### qRT PCR

Primers were designed by Primer-BLAST (NCBI) and ordered from Sigma Proligo (Castle Hill, NSW, Australia) with sequences as follows: PSA (f)5′-agtgcgagaagcattcccaac-3′, (r)5′-ccagcaagatcacgcttttgtt-3′; CD9 (f)5′-ccccaagaaggacgtactcg-3′, (r)5′-gccaaatatcatgaccac ggc-3′; Alix (f)5′-tactctccccaaggaggtgt-3′, (r)5′-tctgctgcatgctg taacctt-3′; TSG101 (f)5′-acagtcagacttgttggggc-3′, (r)5′-gttgcct ggtatggcggata-3′; CD63 (f)5′-cccttggaattgcttttgtcg-3′, (r)5′-cg tagccacttctgatactcttc-3′; CD81 (f)5′-cagaccaccaacctcctgtat-3′, (r)5′-gattcctggatggccccgta-3′; AR (f)5′-ctggacacgacaacaac cag-3′, (r)5′-cagatcaggggcgaagtaga-3′; and RPL32 (f)5′-gcaccaccagtcagaccgatatg-3′, (r)5′-actgggcagcatgtgctttg-3′.

Gene expression was normalized to the housekeeping gene rpl32, and then expressed relative to the vehicle control at the same time point. Data were analysed with SDS 2.3 software by means of the 2-ΔΔCt method [81]. RNA was isolated using RNeasy (Qiagen, Hilden, Germany) according to the manufacturer’s instructions at the end of the experiments. Statistical analysis was performed using Student T-test (Excel). Significant differences in gene expression between two groups were identified with p ≤ 0.05.

### Plasma collection

Plasma from men with prostate cancer (n = 6) was isolated at the Epworth Hospital, Melbourne after written consent (Ethics Approval Epworth Study no.: 34506). Plasma samples from men were collected before digital rectal exam (DRE) at the time of day most convenient to the patient. For inclusion in the study, PSA levels and prostate biopsy results had to meet or exceed the following clinic-pathological criteria (PSA> 20ng/mL, Gleason pattern 4 or above (Gleason 7 (4+3) up to 9 (4+5)), clinical stage >/= T2c). All patients were treatment naïve, had had no detectable bony metastatic disease and no history of other malignancies for the previous 5 years. Plasma from men with benign prostate hyperplasia (n = 10) was collected by Australian Prostate Cancer Collaboration (APCC) Bioresource (after written consent, Ethics approval no.: 1000001165) and used as controls. Blood was collected from patients and processed fresh. Using 10-mL serum tubes with clot activator (Vacutainer 367820; Becton Dickinson), serum was also isolated. The blood was allowed to clot at room temperature for 1 h. Plasma was prepared by centrifugation at 2000 ×g for 10 min at 25°C. The plasma supernatant was removed by pipette, leaving 0.5 cm to avoid disturbing the serum–clot interface and stored at −80°C.

### EV measurement using time resolved-fluorescence immuno assay (TR-FIA)

Conditioned media were collected at the end of each experiment and spun at 2,000 ×g for 20 min to eliminate dead cells and debris and stored at −80°C. Samples (150 μg protein) for cell conditioned medium were diluted in PBS and measured using CD9 or CD63 TR-FIA plates (Cell Guidance System, Carlsbad, USA) for three biological replicates, each in duplicate. Ratio of mean ± SE was calculated and statistical analysis was performed using Student T-test (Excel). Plasma samples (2500 μg protein) were diluted in PBS and measured in duplicate. Ratio of mean ± SD was calculated and statistical analysis was performed between groups using Student T-test (Excel). Significant differences between two groups were identified with p ≤ 0.05.

### Confocal microscopy

Cells were seeded onto coverslips, fixed at 48 h after DHT treatment, labelled with anti-CD9 antibody (1:200, Santa Cruz), followed by secondary anti-mouse fluorescent probe Alexa Fluor 488 (1:500, Molecular Probes, Eugene, US) together with phalloidin-Alexa Fluor 647 (1:50, Invitrogen, Waltham, US) as described [81]. Fixed specimens were imaged using a Zeiss Meta 510 confocal laser-scanning microscope, with a 63X/1.40 Oil DIC M27 objective lens.

## SUPPLEMENTARY MATERIALS FIGURES AND TABLES







## References

[1] Heinlein CA, Chang C (2004). Androgen receptor in prostate cancer. Endocr Rev.

[2] Cai C, Chen S, Ng P, Bubley GJ, Nelson PS, Mostaghel EA, Marck B, Matsumoto AM, Simon NI, Wang H, Balk SP (2011). Intratumoral de novo steroid synthesis activates androgen receptor in castration-resistant prostate cancer and is upregulated by treatment with CYP17A1 inhibitors. Cancer Res.

[3] Scher HI, Sawyers CL (2005). Biology of progressive, castration-resistant prostate cancer: directed therapies targeting the androgen-receptor signaling axis. J Clin Oncol.

[4] Wyatt AW, Gleave ME (2015). Targeting the adaptive molecular landscape of castration-resistant prostate cancer. EMBO Mol Med.

[5] Soekmadji C, Russell PJ, Nelson CC (2013). Exosomes in prostate cancer: putting together the pieces of a puzzle. Cancers.

[6] Urbanelli L, Magini A, Buratta S, Brozzi A, Sagini K, Polchi A, Tancini B, Emiliani C (2013). Signaling pathways in exosomes biogenesis, secretion and fate. Genes.

[7] Choi DS, Kim DK, Kim YK, Gho YS (2014). Proteomics of extracellular vesicles: Exosomes and ectosomes. Mass spectrometry reviews.

[8] Raposo G, Stoorvogel W (2013). Extracellular vesicles: exosomes, microvesicles, and friends. J Cell Biol.

[9] Valadi H, Ekstrom K, Bossios A, Sjostrand M, Lee JJ, Lotvall JO (2007). Exosome-mediated transfer of mRNAs and microRNAs is a novel mechanism of genetic exchange between cells. Nat Cell Biol.

[10] Hendrix A, Hume AN (2011). Exosome signaling in mammary gland development and cancer. The International journal of developmental biology.

[11] Alderton GK (2012). Metastasis. Exosomes drive premetastatic niche formation. Nat Rev Cancer.

[12] Somasundaram R, Herlyn M (2012). Melanoma exosomes: messengers of metastasis. Nat Med.

[13] Wolfers J, Lozier A, Raposo G, Regnault A, Thery C, Masurier C, Flament C, Pouzieux S, Faure F, Tursz T, Angevin E, Amigorena S, Zitvogel L (2001). Tumor-derived exosomes are a source of shared tumor rejection antigens for CTL cross-priming. Nat Med.

[14] Valenti R, Huber V, Iero M, Filipazzi P, Parmiani G, Rivoltini L (2007). Tumor-released microvesicles as vehicles of immunosuppression. Cancer Res.

[15] Lakhal S, Wood MJ (2011). Exosome nanotechnology: an emerging paradigm shift in drug delivery: exploitation of exosome nanovesicles for systemic in vivo delivery of RNAi heralds new horizons for drug delivery across biological barriers. Bioessays.

[16] Escudier B, Dorval T, Chaput N, Andre F, Caby MP, Novault S, Flament C, Leboulaire C, Borg C, Amigorena S, Boccaccio C, Bonnerot C, Dhellin O (2005). Vaccination of metastatic melanoma patients with autologous dendritic cell (DC) derived-exosomes: results of thefirst phase I clinical trial. J Transl Med.

[17] Melo SA, Luecke LB, Kahlert C, Fernandez AF, Gammon ST, Kaye J, LeBleu VS, Mittendorf EA, Weitz J, Rahbari N, Reissfelder C, Pilarsky C, Fraga MF (2015). Glypican-1 identifies cancer exosomes and detects early pancreatic cancer. Nature.

[18] Tkach M, Thery C (2016). Communication by Extracellular Vesicles: Where We Are and Where We Need to Go. Cell.

[19] Thery C, Zitvogel L, Amigorena S (2002). Exosomes: composition, biogenesis and function. Nat Rev Immunol.

[20] Subra C, Laulagnier K, Perret B, Record M (2007). Exosome lipidomics unravels lipid sorting at the level of multivesicular bodies. Biochimie.

[21] Ratajczak J, Wysoczynski M, Hayek F, Janowska-Wieczorek A, Ratajczak MZ (2006). Membrane-derived microvesicles: important and underappreciated mediators of cell-to-cell communication. Leukemia.

[22] Cocucci E, Meldolesi J (2015). Ectosomes and exosomes: shedding the confusion between extracellular vesicles. Trends Cell Biol.

[23] Akers JC, Gonda D, Kim R, Carter BS, Chen CC (2013). Biogenesis of extracellular vesicles (EV): exosomes, microvesicles, retrovirus-like vesicles, and apoptotic bodies. J Neurooncol.

[24] Lima LG, Chammas R, Monteiro RQ, Moreira ME, Barcinski MA (2009). Tumor-derived microvesicles modulate the establishment of metastatic melanoma in a phosphatidylserine-dependent manner. Cancer Lett.

[25] Kim J, Morley S, Le M, Bedoret D, Umetsu DT, Di Vizio D, Freeman MR (2014). Enhanced shedding of extracellular vesicles from amoeboid prostate cancer cells: potential effects on the tumor microenvironment. Cancer Biol Ther.

[26] Uhlen M, Bjorling E, Agaton C, Szigyarto CA, Amini B, Andersen E, Andersson AC, Angelidou P, Asplund A, Asplund C, Berglund L, Bergstrom K, Brumer H (2005). A human protein atlas for normal and cancer tissues based on antibody proteomics. Mol Cell Proteomics.

[27] Bobrie A, Thery C (2013). Exosomes and communication between tumours and the immune system: are all exosomes equal?. Biochem Soc Trans.

[28] Murayama Y, Shinomura Y, Oritani K, Miyagawa J, Yoshida H, Nishida M, Katsube F, Shiraga M, Miyazaki T, Nakamoto T, Tsutsui S, Tamura S, Higashiyama S (2008). The tetraspanin CD9 modulates epidermal growth factor receptor signaling in cancer cells. J Cell Physiol.

[29] Li W, Tait JF (1998). Regulatory effect of CD9 on calcium-stimulated phosphatidylserine exposure in Jurkat T lymphocytes. Arch Biochem Biophys.

[30] Lee C, Sutkowski DM, Sensibar JA, Zelner D, Kim I, Amsel I, Shaw N, Prins GS, Kozlowski JM (1995). Regulation of proliferation and production of prostate-specific antigen in androgen-sensitive prostatic cancer cells, LNCaP, by dihydrotestosterone. Endocrinology.

[31] Hosseini-Beheshti E, Pham S, Adomat H, Li N, Guns ES (2012). Exosomes as Biomarker Enriched Microvesicles: Characterization of Exosomal Proteins derived from a Panel of Prostate Cell Lines with distinct AR phenotypes. Mol Cell Proteomics.

[32] Jansen FH, Krijgsveld J, van Rijswijk A, van den Bemd GJ, van den Berg MS, van Weerden WM, Willemsen R, Dekker LJ, Luider TM, Jenster G (2009). Exosomal secretion of cytoplasmic prostate cancer xenograft-derived proteins. Mol Cell Proteomics.

[33] Locke JA, Wasan KM, Nelson CC, Guns ES, Leon CG (2008). Androgen-mediated cholesterol metabolism in LNCaP and PC-3 cell lines is regulated through two different isoforms of acyl-coenzyme A: Cholesterol Acyltransferase (ACAT). Prostate.

[34] Bakin RE, Gioeli D, Sikes RA, Bissonette EA, Weber MJ (2003). Constitutive activation of the Ras/mitogen-activated protein kinase signaling pathway promotes androgen hypersensitivity in LNCaP prostate cancer cells. Cancer Res.

[35] Bakin RE, Gioeli D, Bissonette EA, Weber MJ (2003). Attenuation of Ras signaling restores androgen sensitivity to hormone-refractory C4-2 prostate cancer cells. Cancer Res.

[36] Ulrix W, Swinnen JV, Heyns W, Verhoeven G (1998). Identification of the phosphatidic acid phosphatase type 2a isozyme as an androgen-regulated gene in the human prostatic adenocarcinoma cell line LNCaP. J Biol Chem.

[37] Shigemura K, Sung SY, Kubo H, Arnold RS, Fujisawa M, Gotoh A, Zhau HE, Chung LW (2007). Reactive oxygen species mediate androgen receptor- and serum starvation-elicited downstream signaling of ADAM9 expression in human prostate cancer cells. Prostate.

[38] Duijvesz D, Versluis CY, van der Fels CA, Vredenbregt-van den Berg MS, Leivo J, Peltola MT, Bangma CH, Pettersson KS, Jenster G (2015). Immuno-based detection of extracellular vesicles in urine as diagnostic marker for prostate cancer. Int J Cancer.

[39] Alcayaga-Miranda F, Gonzalez PL, Lopez-Verrilli A, Varas-Godoy M, Aguila-Diaz C, Contreras L, Khoury M (2016). Prostate tumor-induced angiogenesis is blocked by exosomes derived from menstrual stem cells through the inhibition of reactive oxygen species. Oncotarget.

[40] Quackenbush JF, Cassidy PB, Pfeffer LM, Boucher KM, Hawkes JE, Pfeffer SR, Kopelovich L, Leachman SA (2014). Isolation of circulating microRNAs from microvesicles found in human plasma. Methods Mol Biol.

[41] Trock BJ (2014). Circulating biomarkers for discriminating indolent from aggressive disease in prostate cancer active surveillance. Curr Opin Urol.

[42] Chuan Y, Pang ST, Bergh A, Norstedt G, Pousette A (2005). Androgens induce CD-9 in human prostate tissue. Int J Androl.

[43] Bobrie A, Colombo M, Krumeich S, Raposo G, Thery C (2012). Diverse subpopulations of vesicles secreted by different intracellular mechanisms are present in exosome preparations obtained by differential ultracentrifugation. Journal of extracellular vesicles.

[44] Soekmadji C, Nelson CC (2015). The Emerging Role of Extracellular Vesicle-Mediated Drug Resistance in Cancers: Implications in Advanced Prostate Cancer. Biomed Res Int.

[45] Oren I, Fleishman SJ, Kessel A, Ben-Tal N (2004). Free diffusion of steroid hormones across biomembranes: a simplex search with implicit solvent model calculations. Biophys J.

[46] Ifon ET, Pang AL, Johnson W, Cashman K, Zimmerman S, Muralidhar S, Chan WY, Casey J, Rosenthal LJ (2005). U94 alters FN1 and ANGPTL4 gene expression and inhibits tumorigenesis of prostate cancer cell line PC3. Cancer Cell Int.

[47] Suetens A, Moreels M, Quintens R, Chiriotti S, Tabury K, Michaux A, Gregoire V, Baatout S (2014). Carbon ion irradiation of the human prostate cancer cell line PC3: a whole genome microarray study. Int J Oncol.

[48] Wei X, Du ZY, Zheng X, Cui XX, Conney AH, Zhang K (2012). Synthesis and evaluation of curcumin-related compounds for anticancer activity. European journal of medicinal chemistry.

[49] Cook GA, Longhurst CM, Grgurevich S, Cholera S, Crossno JT, Jennings LK (2002). Identification of CD9 extracellular domains important in regulation of CHO cell adhesion to fibronectin and fibronectin pericellular matrix assembly. Blood.

[50] Longhurst CM, Jacobs JD, White MM, Crossno JT, Fitzgerald DA, Bao J, Fitzgerald TJ, Raghow R, Jennings LK (2002). Chinese hamster ovary cell motility to fibronectin is modulated by the second extracellular loop of CD9. Identification of a putative fibronectin binding site. J Biol Chem.

[51] Ovalle S, Gutierrez-Lopez MD, Olmo N, Turnay J, Lizarbe MA, Majano P, Molina-Jimenez F, Lopez-Cabrera M, Yanez-Mo M, Sanchez-Madrid F, Cabanas C (2007). The tetraspanin CD9 inhibits the proliferation and tumorigenicity of human colon carcinoma cells. Int J Cancer.

[52] Schuurmans AL, Bolt J, Veldscholte J, Mulder E (1991). Regulation of growth of LNCaP human prostate tumor cells by growth factors and steroid hormones. J Steroid Biochem Mol Biol.

[53] Yuan X, Li T, Wang H, Zhang T, Barua M, Borgesi RA, Bubley GJ, Lu ML, Balk SP (2006). Androgen receptor remains critical for cell-cycle progression in androgen-independent CWR22 prostate cancer cells. Am J Pathol.

[54] Kirschenbaum A, Ren M, Levine AC (1993). Enhanced androgen sensitivity in serum-free medium of a subline of the LNCaP human prostate cancer cell line. Steroids.

[55] Lu S, Tsai SY, Tsai MJ (1999). Molecular mechanisms of androgen-independent growth of human prostate cancer LNCaP-AI cells. Endocrinology.

[56] Heijnen HF, Schiel AE, Fijnheer R, Geuze HJ, Sixma JJ (1999). Activated platelets release two types of membrane vesicles: microvesicles by surface shedding and exosomes derived from exocytosis of multivesicular bodies and alpha-granules. Blood.

[57] Morita E, Sandrin V, Chung HY, Morham SG, Gygi SP, Rodesch CK, Sundquist WI (2007). Human ESCRT and ALIX proteins interact with proteins of the midbody and function in cytokinesis. EMBO J.

[58] Tomas A, Vaughan SO, Burgoyne T, Sorkin A, Hartley JA, Hochhauser D, Futter CE (2015). WASH and Tsg101/ALIX-dependent diversion of stress-internalized EGFR from the canonical endocytic pathway. Nat Commun.

[59] Raiborg C, Malerod L, Pedersen NM, Stenmark H (2008). Differential functions of Hrs and ESCRT proteins in endocytic membrane trafficking. Exp Cell Res.

[60] Colombo M, Moita C, van Niel G, Kowal J, Vigneron J, Benaroch P, Manel N, Moita LF, Thery C, Raposo G (2013). Analysis of ESCRT functions in exosome biogenesis, composition and secretion highlights the heterogeneity of extracellular vesicles. J Cell Sci.

[61] Sun Z, Pan J, Bubley G, Balk SP (1997). Frequent abnormalities of TSG101 transcripts in human prostate cancer. Oncogene.

[62] Felten A, Brinckmann D, Landsberg G, Scheidtmann KH (2013). Zipper-interacting protein kinase is involved in regulation of ubiquitination of the androgen receptor, thereby contributing to dynamic transcription complex assembly. Oncogene.

[63] Burgdorf S, Leister P, Scheidtmann KH (2004). TSG101 interacts with apoptosis-antagonizing transcription factor and enhances androgen receptor-mediated transcription by promoting its monoubiquitination. J Biol Chem.

[64] Sun Z, Pan J, Hope WX, Cohen SN, Balk SP (1999). Tumor susceptibility gene 101 protein represses androgen receptor transactivation and interacts with p300. Cancer.

[65] Lee YG, Korenchuk S, Lehr J, Whitney S, Vessela R, Pienta KJ (2001). Establishment and characterization of a new human prostatic cancer cell line: DuCaP. In vivo.

[66] Sfanos KS, Aloia AL, Hicks JL, Esopi DM, Steranka JP, Shao W, Sanchez-Martinez S, Yegnasubramanian S, Burns KH, Rein A, De Marzo AM (2011). Identification of replication competent murine gammaretroviruses in commonly used prostate cancer cell lines. PLoS One.

[67] Knouf EC, Metzger MJ, Mitchell PS, Arroyo JD, Chevillet JR, Tewari M, Miller AD (2009). Multiple integrated copies and high-level production of the human retrovirus XMRV (xenotropic murine leukemia virus-related virus) from 22Rv1 prostate carcinoma cells. J Virol.

[68] Sato K, Aoki J, Misawa N, Daikoku E, Sano K, Tanaka Y, Koyanagi Y (2008). Modulation of human immunodeficiency virus type 1 infectivity through incorporation of tetraspanin proteins. J Virol.

[69] van Dongen HM, Masoumi N, Witwer KW, Pegtel DM (2016). Extracellular Vesicles Exploit Viral Entry Routes for Cargo Delivery. Microbiol Mol Biol Rev.

[70] Brasset E, Taddei AR, Arnaud F, Faye B, Fausto AM, Mazzini M, Giorgi F, Vaury C (2006). Viral particles of the endogenous retrovirus ZAM from Drosophila melanogaster use a pre-existing endosome/exosome pathway for transfer to the oocyte. Retrovirology.

[71] Levina E, Ji H, Chen M, Baig M, Oliver D, Ohouo P, Lim CU, Schools G, Carmack S, Ding Y, Broude EV, Roninson IB, Buttyan R (2015). Identification of novel genes that regulate androgen receptor signaling and growth of androgen-deprived prostate cancer cells. Oncotarget.

[72] Duijvesz D, Versluis CY, van der Fels CA, Vredenbregt-van den Berg MS, Leivo J, Peltola MT, Bangma CH, Pettersson KS, Jenster G (2015). Immuno-based detection of extracellular vesicles in urine as diagnostic marker for prostate cancer. Int J Cancer.

[73] Thery C, Amigorena S, Raposo G, Clayton A (2006). Isolation and characterization of exosomes from cell culture supernatants and biological fluids. Curr Protoc Cell Biol.

[74] Inder KL, Loo D, Zheng YZ, Parton RG, Foster LJ, Hill MM (2012). Normalization of protein at different stages in SILAC subcellular proteomics affects functional analysis. Journal of Integrated Omics.

[75] Nesvizhskii AI, Keller A, Kolker E, Aebersold R (2003). A statistical model for identifying proteins by tandem mass spectrometry. Analytical chemistry.

[76] Ishihama Y, Oda Y, Tabata T, Sato T, Nagasu T, Rappsilber J, Mann M (2005). Exponentially modified protein abundance index (emPAI) for estimation of absolute protein amount in proteomics by the number of sequenced peptides per protein. Mol Cell Proteomics.

[77] Hohn O, Krause H, Barbarotto P, Niederstadt L, Beimforde N, Denner J, Miller K, Kurth R, Bannert N (2009). Lack of evidence for xenotropic murine leukemia virus-related virus(XMRV) in German prostate cancer patients. Retrovirology.

[78] Sadowski MC, Pouwer RH, Gunter JH, Lubik AA, Quinn RJ, Nelson CC (2014). The fatty acid synthase inhibitor triclosan: repurposing an anti-microbial agent for targeting prostate cancer. Oncotarget.

[79] Barreiro O, Yanez-Mo M, Sala-Valdes M, Gutierrez-Lopez MD, Ovalle S, Higginbottom A, Monk PN, Cabanas C, Sanchez-Madrid F (2005). Endothelial tetraspanin microdomains regulate leukocyte firm adhesion during extravasation. Blood.

[80] Livak KJ, Schmittgen TD (2001). Analysis of relative gene expression data using real-time quantitative PCR and the 2-[Delta][Delta] CT method. Methods.

[81] Jang Y, Soekmadji C, Mitchell JM, Thomas WG, Thorn P (2012). Real-time measurement of F-actin remodelling during exocytosis using Lifeact-EGFP transgenic animals. PLoS One.

