# Supplementary material for: Modulation of paracrine signaling by CD9 positive small extracellular vesicles mediates cellular growth of androgen deprived prostate cancer
Source: Oncotarget. 2016 Aug 8;8(32):52237–55. doi: 10.18632/oncotarget.11111 (PMC5581025; doi:10.18632/oncotarget.11111)
Supplement: Supplementary file 1 [file oncotarget-08-52237-s001.pdf]

# Modulation of paracrine signaling by CD9 positive small extracellular vesicles mediates cellular growth of androgen deprived prostate cancer

## SUPPLEMENTARY FIGURES AND TABLES

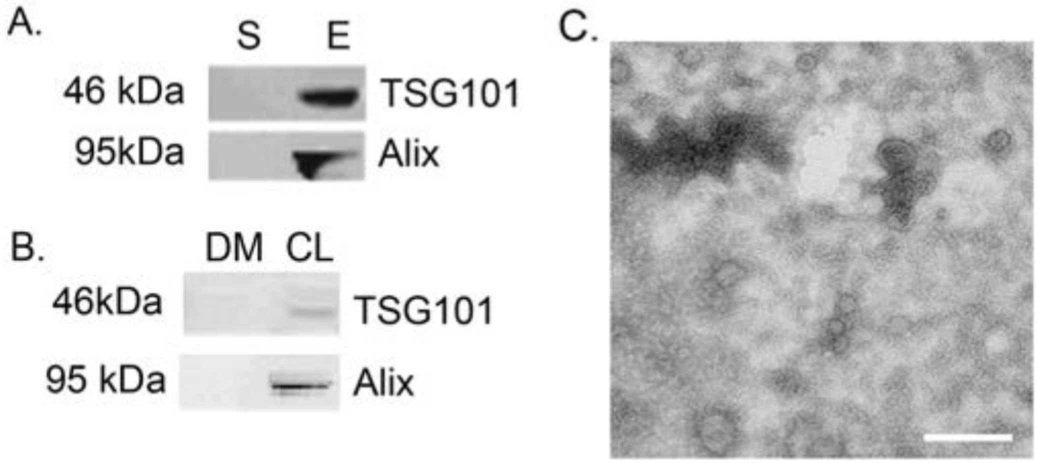


**Supplementary Figure S1: EV can be isolated from conditioned medium of LNCaP in androgen deprived condition. A.** Western blot illustrates Alix & TSG101 in the EV pellet. S: supernatant after PBS washing step 100,000 g spin for 1h 30min, E: final pellet (10µg). **B.** Western blot illustrates that TSG101 and Alix were not found in the pre-cleared 5% serum depleted medium used to collect cell line derived EV. DM indicates 5% CSS EV depleted medium; CL indicates cell lysates used as a positive control. **C.** TEM image of EV isolated from androgen deprived LNCaP cells (grown in CSS). Scale bar i=200 nm.

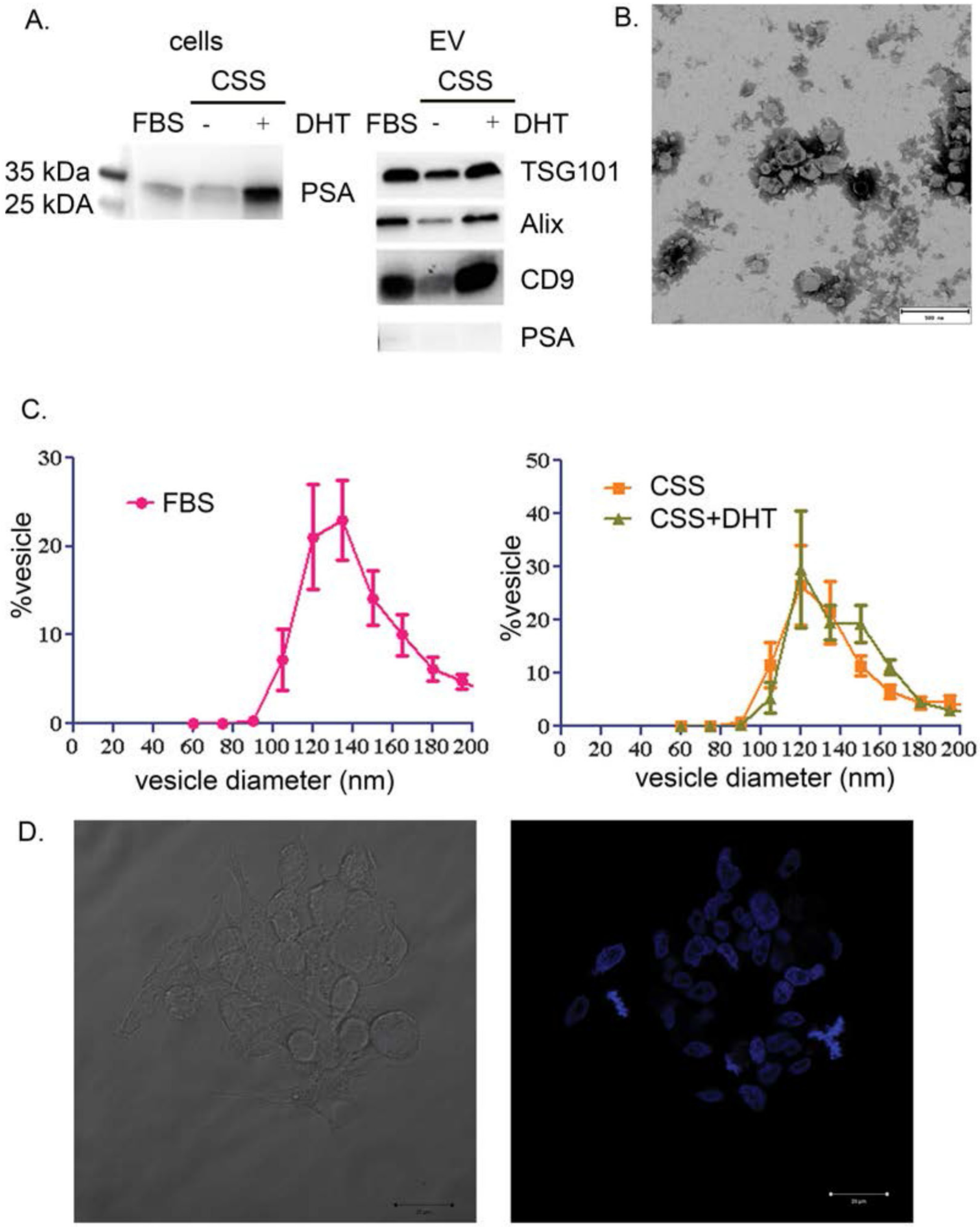


**Supplementary Figure S2: EV secreted by DUCaP cells. A.** A representative of western blot image illustrates the expression of EV markers found in DUCaP cell lysates (30 µg) or EV (10 µg). Common EV markers TSG101, Alix, and CD9 were found in DUCaP EV, as well as CHC, but not PSA. **B.** A representative image of EV secreted by DUCaP cells (scale bar i=500nm) **C.** The diameters of vesicles secreted by DUCaP cells grown in FBS (+ EtOH, vehicle), CSS (+ EtOH, vehicle) and CSS+10nM DHT were measured by qNANO. There were no significant changes on the vesicles size across treatments. Data were represented as mean +/- SEM. **D.** Morphology of fibroblast-free DUCaP cells, nuclei are stained with DAPI (blue). Cells were fixed in 4% paraformaldehyde in PBS for 15 minutes, and stained with DAPI. Coverslips were washed in PBS and mounted using Prolong Gold Antifade (Invitrogen). Fixed specimens were imaged using a Zeiss Meta 510 confocal laser scanning microscope, with a 63X/1.40 Oil DIC M27 objective lens.

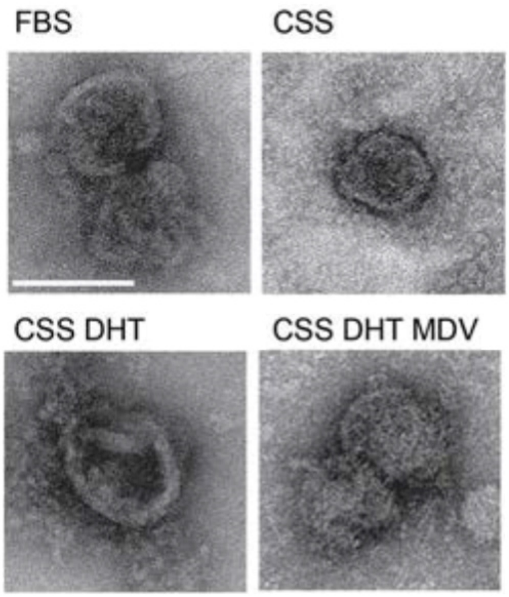


**Supplementary Figure S3: Representative images captured by Transmission Electron Microscopy of vesicles secreted by LNCaP cells (scale bar: i=100nm).** LNCaP cells were grown in FBS (+ EtOH), CSS (+ EtOH), CSS+10 nM DHT and CSS+10 nM DHT+10 µM MDV3100.

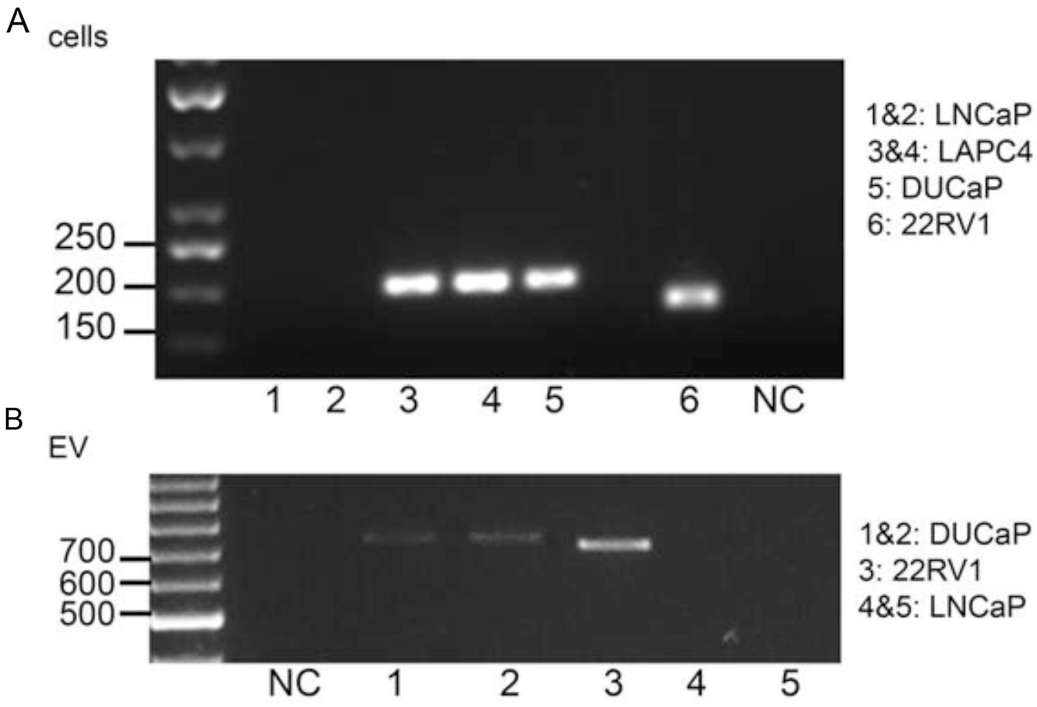


**Supplementary Figure S4: Detection of Murine leukemia virus-related virus (MuLV) in PCa cell lines and extracellular vesicles.** Sequence comparisons demonstrate that the nested primer set are able to detect a number of different MuLV-like viruses, including those previously isolated from xenograft derived-cell lines, including LAPC4 (JF908816.1), VCaP (JF908815.1) and 22Rv1 (FN692043.2). **A.** RNA corresponding to murine leukaemia viruses could be detected by nested PCR and isolated from LAPC4 (lane 3 and 4) and 22Rv1 (lane 6) cells. Both cells are known to harbour MuLV-like viruses. The same band was observed in DUCaP (lane 5) cells, but not LNCaP cells (lane 1 and 2) or negative control (NC, $H_2O$). **B.** RNA sequences for MuLV-like viruses could also be detected by PCR in EV isolated from DUCaP (lane 1 and 2) and 22Rv1 cells (lane 3), but not LNCaPs (lanes 4 and 5).

**Supplementary Table S1: eMPAI ratio of common proteins found in extracellular vesicles isolated from LNCaP and DUCaP grown in CSS (+ EtOH) and CSS+DHT.**

**See Supplementary File 1**

**Supplementary Table S2: List of DHT regulated genes (AR regulated genes, ARG).** To establish a comprehensive signature of androgen responsive genes in prostate cancer cells, we have combined data from 4 independent unpublished in-house microarray experiments where cultured LNCaP cells were treated with 10 nM DHT for 48 hrs. Each experiment contained at least three repeats of every sample and was processed through the same pipeline using our custom-designed prostate cancer focused 180k Agilent oligo microarray. For each dataset, a quantile between array normalization was applied and differential expression was determined using the Baysian adjusted t-statistic linear model of the 'Linear Models for Microarray Data' (LIMMA) package in R. The p-values were corrected for a false discovery rate of 5% and probes with an adjusted p-value of <=0.05 and an average fold change of >=1.5 were considered as differentially expressed. To collate probe lists into gene lists, the differentially expressed RefSeq probe with the highest fold change was chosen to represent the gene. The 342 genes common between all 4 generated lists and showing the same directionality of regulation in all 4 datasets represent the final gene signature. Microarray experiments were performed on a custom 180k Agilent oligo microarray (VPCv3, ID032034, GPL16604) as described in Shieh et al. 2012 (Plos One).

**See Supplementary File 2**
